# Clarifying the mechanisms and resources that enable the reciprocal involvement of seldom heard groups in health and social care research: A collaborative rapid realist review process

**DOI:** 10.1111/hex.12865

**Published:** 2019-02-06

**Authors:** Éidín Ní Shé, Sarah Morton, Veronica Lambert, Cliona Ní Cheallaigh, Vanessa Lacey, Eleanor Dunn, Cliona Loughnane, Joan O'Connor, Amanda McCann, Maura Adshead, Thilo Kroll

**Affiliations:** ^1^ School of Nursing, Midwifery and Health Systems University College Dublin Dublin Ireland; ^2^ School of Social Policy, Social Work and Social Justice University College Dublin Dublin Ireland; ^3^ School of Nursing and Human Sciences Dublin City University Dublin Ireland; ^4^ Consultant in General Medicine and Infectious Diseases St James's Hospital Dublin Ireland; ^5^ School of Medicine Trinity College Dublin Dublin Ireland; ^6^ Transgender Equality Network Ireland Dublin Ireland; ^7^ Dublin Simon Community Dublin Ireland; ^8^ National Women's Council of Ireland Dublin Ireland; ^9^ Disability Federation of Ireland Dublin Ireland; ^10^ UCD Conway Institute of Biomolecular and Biomedical Science and UCD School of Medicine University College Dublin UCD Dublin Ireland; ^11^ School of Politics and Public Administration University of Limerick Limerick Ireland

**Keywords:** behaviour change wheel, co‐design, health and social care research, public and patient involvement, rapid realist review, seldom heard

## Abstract

**Objective:**

Public and patient involvement is increasingly embedded as a core activity in research funding calls and best practice guidelines. However, there is recognition of the challenges that prevail to achieve genuine and equitable forms of engagement. Our objective was to identify the mechanisms and resources that enable the reciprocal involvement of seldom heard groups in health and social care research.

**Methods:**

A rapid realist review of the literature that included: (a) a systematic search of CINAHL, PsycINFO, PubMed and Open Grey (2007‐2017); (b) documents provided by expert panel members of relevant journals and grey literature. Six reference panels were undertaken with homeless, women's, transgender, disability and Traveller and Roma organizations to capture local insights. Data were extracted into a theory‐based grid linking context to behaviour change policy categories.

**Main results:**

From the review, 20 documents were identified and combined with the reference panel summaries. The expert panel reached consensus about 33 programme theories. These relate to environmental and social planning (7); service provision (6); guidelines (4); fiscal measures (6); communication and marketing (4); and regulation and legislation (6).

**Conclusions:**

While there is growing evidence of the merits of undertaking PPI, this rarely extends to the meaningful involvement of seldom heard groups. The 33 programme theories agreed by the expert panel point to a variety of mechanisms and resources that need to be considered. Many of the programme theories identified point to the need for a radical shift in current practice to enable the reciprocal involvement of seldom heard groups.

## 1 INTRODUCTION

While there is no consensus on one definition for public and patient involvement (PPI) nor in relation to the terminology used (e.g engagement and involvement are often used interchangeably or with different connotations), there is a growing abundance of academic and grey literature on the merits, impact and experiences of PPI in health and social care research.^1^, ^2^, ^3^, ^4^, ^5^ In the UK, the National Institute for Health Research (NIHR) has set up the platform INVOLVE to promote and share best practices for PPI. In Canada, the Institute for Health Research (CIHR) has developed a strategy for Patient Outcome Research (POR). While in Ireland, the Health Research Board (HRB) launched the PPI Ignite Awards in 2017 which are focused on enabling institutional‐wide PPI responses within universities. As PPI becomes more embedded as a core activity in many national and international funding calls, the evaluative literature has shifted to capture impacts.^6^, ^7^, ^8^ A recent systematic review and modified Delphi process to capture an agreement on the principles underpinning PPI point to a new focus on what is required to sustain and embed these principles within university structures.^9^ More recently, Palmer et al^10^ have provided a welcome depth to the theory on the processes of co‐production and co‐design within mental health improvement and system redesign. These shifts are to be welcomed but caution and recognition of the challenges are also prevalent in the recent literature.^11^, ^12^, ^13^, ^14^, ^15^ In particular, there is a recognition that PPI partners’ involvement can be tokenistic often at the lower level of consultation.^1^ Ocloo and Matthews outline a range of reasons why achieving genuine patient involvement presents challenges, citing in particular a lack of diversity of those becoming involved. They call for the inclusion of more diverse populations via the implementation of more inclusive and democratic models of engagement that are embedded in co‐design.^11^

The merits of overcoming the often‐identified challenges of engaging diverse voice or “seldom heard” groups have been stressed where different perspectives informed by, for example, socio‐economic status, ethnicity, health status or gender can provide deeper insights in designing and implementing a trial.^16^ There is also a recognition in the literature of the challenges of engaging diverse populations. These engagements often occur at the lowest levels focused on consulting rather than involving.^11^, ^12^ The demands on researchers to involve more diverse populations and to move to higher levels of co‐produced involvement bring new demands to support, develop and sustain. Key to this is to understand and map efforts, initiatives and strategies designed to enhance the collaborative capacity skills of researchers, the public and those working within the health system.^14^, ^15^, ^16^ The focus of this paper was to identify the strategies that may help overcome the often‐identified challenges of engaging seldom heard groups. Being seldom heard means that existing structures and processes in organizations including universities and health and social care providers may not be adequately matched to the needs of all members of the public. The aim for this rapid realist review (RRR) was to clarify the mechanisms and resources required to enable seldom heard people to be involved in health and social care research. A characterization of seldom heard has been provided in a protocol paper published prior to the review.^17^

## 2 METHODS

### 2.1 Study methods

An RRR approach was chosen as it explicitly allows for the engagement of knowledge users throughout the review process.^17^ In contrast to a systematic review, the RRR builds an understanding of why and how things work (programme theories). A detailed rationale for and characterization of the review process has been outlined in our protocol paper.^18^ The RRR adhered to the RAMESES realist publication standards guides with adaptations to streamline and accelerate the process as advised in the literature.^19^, ^20^

### 2.2 Establishment of an expert panel

An expert panel convened in March 2018 consisting of members who have experience in health and social care systems, PPI, co‐design, emancipatory research and people and organizations representing seldom heard groups (Appendix S1). All expert panel members are co‐authors of this paper. The first meeting clarified the scope and the overarching RRR question as being “What are the mechanisms that enable the reciprocal involvement of seldom heard groups?” Key terms were defined and agreed by the group (Appendix S2). The search strategy, conditions and participants were reviewed (Table 1), and the inclusion and exclusion criteria were developed.

**Table 1:** Literature search strategy

**Database Search:** Cumulative Index of Nursing and Allied Health Literature (CINAHL), Psychological Information Database (PsycINFO), PubMed and Open Grey. **String 1 Participants:** Seldom Heard, Fail to Engage, Hard to Reach, Mutually Excluded, Seldom Excluded, Hardly Reached. **String 2 Conditions**: Co‐Design, Co‐Production, Emancipatory Research, Action Research, Reciprocal Involvement, Co‐Creation.

The expert panel created an extraction template (Appendix S3) to ensure that mechanisms and resources would be captured. The expert panel's initial discussion focused on the importance of developing policy responses that could be implemented from the review within their respective organizations. It was therefore agreed that the template would extract contexts linked to adopted policy categories as noted in the behaviour change wheel (BCW).^21^ The linking to the BCW policy categories was used in the template to examine the developed programme theories by providing a contextual overview. The BCW is a recent but increasingly popular taxonomy to assist the development and implementation of behaviour change interventions.^22^ In this review, the BCW was used to describe the mechanisms and related resources of involving seldom heard groups in relation to specific contextual factors.

### 2.3 Reference panel process

Reference panels are local sounding boards undertaken in an RRR to ensure that the review and the developed programme theories are inclusive to the experience of those “on the ground.”^18^ The expert panel identified organizations representing diverse seldom heard people who were to be consulted with via their preferred forum, either face‐to‐face, by phone or via email. Participating organizations were identified following a review by the expert panel of the seldom heard definition (Appendix S2). An open invitation was sent out by the community organizations inviting their members to be involved in the reference panel process. Four questions were created by the expert panel to capture the organizations identified mechanisms and resources (Appendix S4). These would be synthesized to contribute to the RRR programme theories. Six reference panels were consulted in total with the following:

1. Dublin Simon Community: an organization working to prevent and address homelessness in the Dublin, Kildare, Wicklow and Meath.
2. Disability Federation of Ireland (DFI): a 130‐member organization working towards equality for people with disabilities.
3. Pavee Point: a national organization focused on improving the human rights of Irish Travellers and members of the Roma community.
4. Transgender Equality Network of Ireland (TENI): a national organization working on improving conditions and advance the rights and equality of transgender people and their families.
5. Centres for Independent Living (CIL): a national organization enabling independent living for people with disabilities.
6. Longford Women's Network: a women's centre based on the rural midlands town of Longford supporting women to fulfil their potential in a safe and equal society

### 2.4 Data extraction and analysis

Data were extracted from March to June 2018 with fortnightly meetings to critically appraise, analyse and synthesize the data using a data extraction tool (Appendix S3). All extractions undertaken by expert panel members were reviewed by the synthesis lead (ÉNS) and transferred to an extraction table (Appendix S5). Reference panels were conducted from May to July by ÉNS and TK and were summarized into an extraction table (Appendix S6). A final consensus meeting was held by the expert panel in July 2018 to agree on the programme theories.

## 3 RESULTS

### 3.1 Nature of data set

**Figure 1:**
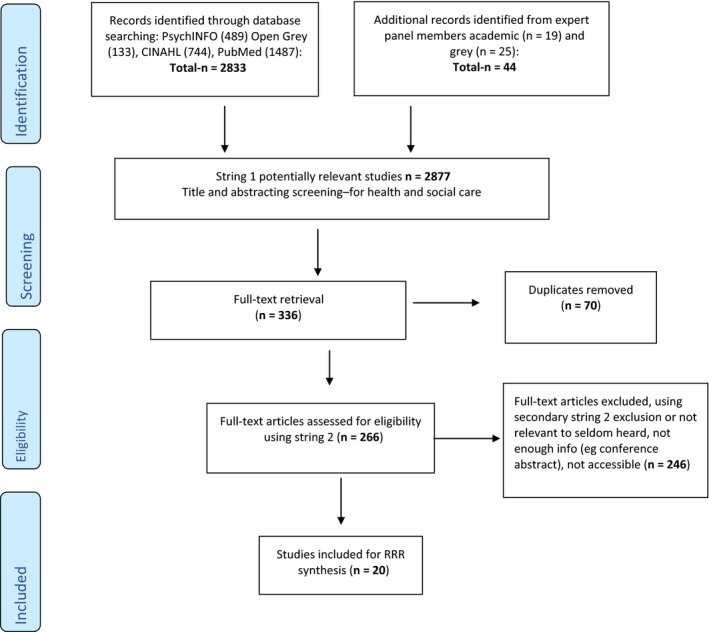
Modified PRISMA flow diagram of data search

After screening and comparison with inclusion/exclusion criteria (Table 2), the final review and synthesis consisted of 20 documents (Figure 1) consisting of the following (Appendix S5 for a summary of all the papers):

**Table 2:** Literature inclusion and exclusion criteria

**Primary Exclusion Criteria** Studies not written in English. Studies that include participants who are not human. Studies that are letters, notes, conference abstracts or reviews only. **Secondary Exclusion Criteria** Studies without descriptions of any intervention or mechanism. Studies that do not report any outcome or result. Studies without health and social research elements. Unable to obtain further information to make assessment. **Inclusion Criteria** Both quantitative and qualitative studies. Both published and grey literature (e.g websites, reports, dissertations and theses). Time frame: 2007‐2017^a^ Health and social care research.

a Expert panel may provide papers outside this time frame.

1. Systematic reviews: Three systematic reviews including an Australian‐based review on barriers to the participation of socio‐economically disadvantaged groups in health research and strategies on how to increase engagement.^23^ The second was a UK‐based review focused on black and minority ethnic group‐PPI involvement in health and social care research.^24^ A UK‐based evidence synthesis was the third review concentrated on health and social interventions for inclusion health for people with experiences of homelessness, drug use, imprisonment and sex work.^25^
2. Empirical articles: Eight in total including a UK study presenting how seldom heard groups and social care services establish inclusive involvement practice.^26^ The second was a Canadian article on engaging frail older people and caregivers in research and decision making.^27^ The third was a US study outlining what worked in engaging diverse non‐English speaking communities.^28^ The fourth was an Irish paper on undertaking participatory learning and action approach in developing GP communication guidelines with migrants.^29^ The fifth was a US study on what worked in developing an educational programme on breast cancer screening amongst African American women.^30^ The sixth was an Australian study on how a randomized controlled trial developed an inclusive methodological approach co‐designed by people affected by severe mental illness.^31^ The seventh was a regional Australian study on engaging families in the design of child protection measures.^32^ The eight study was from the UK that explored differences between what seldom heard groups prioritize in health from mainstream views.^33^
3. Case studies: Three in total including a US case study article on how to diversify participants in clinical research;^34^ a UK study presenting participatory action research between patients and emergency department staff to improve palliative care experiences.^35^ The third article presented three case studies from Australia highlighting learnings in research with highly marginalized young people.^36^
4. Reflective articles: Three articles including a reflective article on developing and sustaining an emancipatory research project between Irish, Ugandan, Tanzanian and South African partners;^37^ a UK reflective article on co‐producing research with an intermediary community partner;^38^ a reflective article identifying methods to engage hard to reach patients in patient‐centred outcomes.^39^
5. Grey literature: Three grey literature articles/reports, including an Irish‐based report on what was learned from an emancipatory research partnership involving community and university partners to capture the experiences of Travellers and people with experience of the asylum process in respect to access of public services.^40^ The second was a UK summary of a seminar exploring best practice themes around service user involvement for women facing multiple disadvantages.^41^ The third was an Irish report to capture the process of people in recovery being involved in identifying and conducting their own research using a peer lead approach.^42^

A summary of findings about the extracted articles and reference panels linked to mechanisms and resources is outlined (Appendix S6).

### 3.2 Agreed programme theories linked with behaviour change wheel policy categories

**Figure 2:**
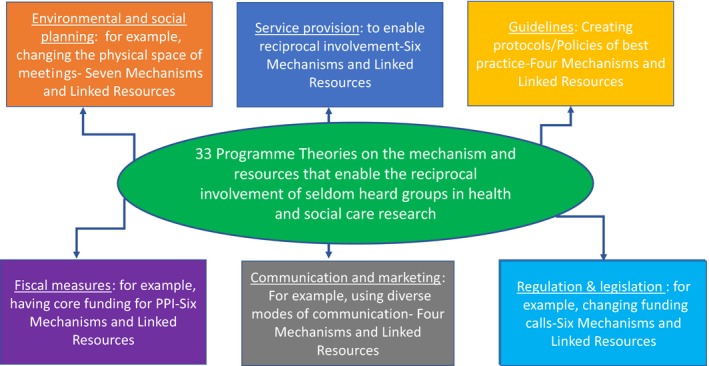
Programme theories linked to behaviour change wheel policy categories

The expert panel reconvened in July 2018 and reviewed the extracted data. The expert panel via consensuses validated and prioritized 33 programme theories linking them to the adopted contextual policy categories as noted in the BCW.^22^ The programme theories were generated from the review and synthesis of findings from the literature, refinement in discussions with reference panels and via the final consensus meeting with the expert panel (Figure 2).

Below is a summary overview of the mechanisms of 33 programme theories (statements on what works) that emerged linked to the six BCW policy categories (Boxes 1, 2, 3, 4, 5, 6).

Box 1 **Programme Theories: Environmental and Social Planning**

1

1. Ensure collaboration and engagement occur in safe, accessible and inclusive spaces as identified by community partners.
2. Enable researcher/s presence in community spaces to develop connections and build trust over time.
3. Undertake an audit of involvement spaces, by all partners, prior to the start of the research project to ensure accessibility and continually monitor with feedback throughout the study.
4. Make available University resources such as access to the library and networking events to community partners; subject to the level of engagement and collaboration.
5. Provide financial resources to community partners to facilitate costs for engagement at community spaces.
6. Share research data and outputs with community partners in an agreed and appropriate way.
7. Provide inclusive women‐only spaces.

Box 2 **Programme Theories: Service Provision**

1

1. Engage community partners to support all co‐production activities before, during and after the research process to enable ongoing feedback.
2. Provide ongoing education to researchers—this should involve active involvement of community partners to support researchers in developing a shared understanding of the social context for which the research is being undertaken.
3. Develop an accredited education programme for community partners that is culturally appropriate to support capacity building.
4. Support the career opportunities and educational progression of community partners.
5. Prioritize consistent and regular follow‐up with community partners that reflect the ongoing needs of community partners.
6. Develop innovative and flexible methods of engagement and outputs with community partners.

Box 3 **Programme Theories: Guidelines**

1

1. Create an engagement/co‐design checklist at the start of the project and assign responsibility amongst partners to review and modify throughout the process.
2. Provide a diversity of involvement options for community partners.
3. Enable flexibility from the start.
4. Develop co‐created guidelines regarding data ownership and usage.

Box 4 **Programme Theories: Fiscal Measures**

1

1. Include costs for psychological supports for researchers, service users and service providers.
2. Include costs for alternative outputs as identified by partners during the research process.
3. Ensure flexibility in payment methods to partner organizations by enabling vouchers or cash when requested.
4. Allocate funding to celebrate success with collaborators to acknowledge the ongoing partnerships.
5. Provide reasonable costs for all community partners’ engagements (e.g food, transport, social and care costs and Personal Assistants).
6. Factor in the time and subsequent resources to develop equitable research partnership.

Box 5 **Programme Theories: Communication and Marketing**

1

1. Allocate time, at the start of the project to allow all partners to articulate what they would like to achieve from the collaboration. This should be written up and agreed upon by all partners.
2. Allocate time, throughout the project, to enable shared decision making in implementing and adapting the study with all partners.
3. Establish a forum for researchers to share their motivations for doing research to overcome any community stereotypes.
4. Fashion research process and community outputs that are accessible and culturally appropriate language using plain English guidelines.

Box 6 **Programme Theories: Regulation and Legislation**

1

1. Review ethics procedures to ensure that the competence of all partners is assumed as the default.
2. Ethics should prioritize a process of ongoing consent.
3. Funding calls need to ensure the time it takes to develop research with seldom heard groups is supported and resourced.
4. Funders should specifically host calls for co‐design/PLA/Emancipatory research.
5. Include operational and budget flexibility in funding calls to enable community partners to identify the supports required during the research process.
6. Funders should consult with community partners in the development and evaluation of research processes and funding calls.

The review found that engagement with seldom heard groups needs to occur in safe, accessible and inclusive spaces.^25^, ^32^, ^33^, ^38^, ^39^, ^40^, ^41^, ^42^ Ensuring spaces are accessible needs to be guaranteed by undertaking an audit at the start of the research, which should be monitored and reviewed throughout the project.^25^, ^40^, ^41^, ^42^ This was referred to in two reference panels with DFI and CIL where participants noted that often they travel to attend meetings in venues which were not accessible. One review article by Adshead and Dubula on undertaking an emancipatory research project between the community and academic partners from Uganda, Tanzania, South Africa and Ireland stressed the importance of making university resources such as libraries and links to networking opportunities available to the community.^37^ Training should also be provided to community members on how to use these university resources. Making funding resources available to community partners to facilitate engagement within community spaces was an important mechanism identified to enable seldom heard participation. This would cover costs for (a) transportation, (b) making food available during activities and (c) having care supports available (e.g childcare and social care).^27^, ^37^, ^38^ Kaiser et al^34^ in particular, outline how a monthly fee was agreed and arranged with community partners to cover such costs. Giving time to develop appropriate data sharing and outputs with all partners as noted by two studies.^23^, ^34^ Having a separate space for engaging seldom heard women was stressed in two grey literature reports, and via the reference panels that should include care and logistic supports.^40^, ^41^

The majority of papers and reference panels stressed that early engagement with community partners was a key enabler to shape the research process from the outset.^25^, ^34^, ^36^, ^37^, ^38^, ^40^ The reference panel with Pavee Point reinforced this point. Often researchers came to the organization with a research project developed which was perceived as culturally inappropriate. Significant time was then spent by the organization reviewing the work which was a considerable source of frustration. TENI also explained how their organization was small and often researchers came to them at the “11th hour” with funding applications which they often reviewed after hours and which took their focus away from other priorities. Providing ongoing education to researchers that includes the active involvement of community partners was stressed. This was seen as important to enable a shared understanding of the broader contexts for which the research is being undertaken.^23^, ^24^, ^33^, ^34^, ^36^, ^37^, ^38^, ^39^ ^,44^ The use of innovative and flexible modes of engagement was also identified.^23^, ^24^, ^25^, ^27^, ^31^, ^35^ ^,45^ Providing pathways to accredited education was noted in four documents.^23^, ^27^, ^37^ ^,44^ The reference panel with Dublin Simon Community highlighted the importance of ensuring that involvement was linked to recognized training, employment support or internship opportunities. Supporting career opportunities and educational progression of community researchers was noted in three articles and was reinforced in the reference panel process.^28^, ^34^, ^40^ Being present with community partners and ensuring feedback is ongoing as agreed with community partners were identified in the reference panels. Ensuring engagement and outputs are flexible and innovative emerged in most of the literature and in the reference panels.^23^, ^24^, ^25^, ^26^, ^27^, ^31^, ^35^, ^36^, ^37^, ^38^, ^39^, ^40^, ^41^, ^42^

The importance of all partners creating and reviewing a co‐design checklist emerged in the Adshead and Dubula study.^37^ They outlined tensions that occurred during the study. This happened between academics who were working towards project timelines agreed by the grant funders and the desire of community partners, who wished to advance emancipatory work at their own pace.^37^ Developing guidelines/protocols were identified as a key mechanism to support diverse involvement, ensuring flexibility and clarity on data ownership and usage.^23^, ^36^

The reference panels with Pavee Point and TENI identified the need to make funding available to include psychological supports as required. The importance of funding for alternative outputs as identified by community partners such as accessible lay summaries was noted in five studies.^24^, ^25^, ^28^, ^34^, ^37^ Flexibility in payments to co‐researchers emerged in the literature and in the reference panels.^23^, ^25^, ^28^, ^34^, ^40^ Dublin Simon Community and DFI reference panels noted that peer researchers could often be in receipt of social welfare payments and research payments could have an impact on this. As such, vouchers, cash or whichever is most appropriate for the community partners were suggested as alternatives. The Kaiser study identified the importance of allocating funding to celebrate success with community partners.^34^ Including funding to cover the cost of involvement emerged as a mechanism in most of the literature.^23^, ^24^, ^25^, ^26^, ^27^, ^28^, ^29^, ^30^, ^31^, ^32^, ^33^, ^34^, ^36^, ^38^, ^39^, ^40^, ^41^ DFI stressed that this was key as often it was expected that they as an organization should cover these costs. Providing resources for the development of a research partnership emerged significantly in Robinson et al,^32^ who also emphasized the need to be present to develop trust.

Identifying what all partners would like to achieve from the study should be prioritized.^37^ The Nguyen et al^28^ study stressed the need to enable time for shared decision making. The Kaiser et al^34^ study noted that community partners often held stereotypical viewpoints about researchers’ motives and pointed to the importance of the research team sharing their motivations for being involved. Ensuring that outputs are accessible and culturally appropriate was stressed by both Pavee Point and Dublin Simon Community representatives.

Two studies identified that university ethics applications and guidelines should be reviewed to always assume competence of study participants and to enable processes to seek verbal consent on an ongoing basis.^34^, ^38^ The time it takes to develop research partnerships should be included in funding calls and specific calls should be focused on co‐design. This was the case in two studies where funding was made available to specifically to undertake emancipatory and participatory action learning approaches.^37^, ^42^ Ensuring flexibility in funding schemes to enable community partners to identify needed supports was identified in the three systematic reviews.^23^, ^24^, ^25^ Finally, the inclusion of community partners in the development and evaluation of funding calls was identified within the reference panel process. The Longford Women's Link also stressed that training for community partners on evaluations be made available to support their capacity to be involved in the process.

## 4 DISCUSSION AND CONCLUSIONS

This RRR process has drawn from diverse literature both grey and empirical supplemented with the insights from the reference panel process and final consensus by the expert panel. To the best of our knowledge, this is the first RRR reviewing the mechanisms and resources that enable the reciprocal involvement of seldom heard people in health and social care research.

The RRR process identified 20 relevant documents and undertook six reference panels with homeless, women's, transgender, disability and Traveller and Roma organizations. The expert panel agreed via consensus programme theories that are statements on what works focusing on the mechanisms and resources to enable the reciprocal involvement of seldom heard groups in health and social research. The expert panel wished to focus the RRR on developing policy responses that could be implemented from the review which was enabled by adopting and linking to the BCW policy categories.^21^ An overarching conclusion from this review was the importance of reciprocity and its role in enabling people to know and control their world by engaging participants from the start of the research project and requires the use of methods such as co‐design, co‐production and emancipatory research (Appendix S2).^6^, ^10^, ^29^, ^31^, ^37^, ^38^, ^39^, ^40^, ^41^, ^42^, ^43^ The 33 programme theories agreed by the expert panel and presented in this paper point to a variety of mechanisms and resources that need to be included to enable the reciprocal involved of seldom heard groups in health and social care research. Many of the programme theories identified are not surprising. They, however, point to the need for a radical shift in current practice to enable the reciprocal involvement of seldom heard groups.

It is recognized through this review of the literature and from our discussions via the reference panel processes that currently undertaking reciprocal PPI with seldom heard groups often requires heroic efforts from all parties involved.^26^, ^31^, ^34^, ^37^, ^38^ Community partners were often enabling research at the 11th hour and spending a lot of time ensuring the project was culturally appropriate and accessible (Appendix S6). Researchers were often working beyond the scope of their funding calls to provide support to their partners and spending significant time in being present with community partners to build relationships and trust.^26^, ^28^, ^30^, ^31^, ^32^, ^33^, ^34^, ^35^, ^36^, ^37^, ^38^, ^39^, ^40^, ^41^, ^42^ The review notes structural challenges that need to be navigated such as ethics, payments and access to university resources for community partners and sustainable funding to enable participation.^23^, ^25^ Having multiple partners working on a project often results in tensions given the remits of different agenda that can emerge.^37^, ^38^ It is important that time and adequate flexible resources are made available to celebrate success and achievements.^32^ The review also found that funders have a key role to play to enable the reciprocal involvement of seldom heard groups.^29^, ^40^ As the shift away from a “fund and forget model” continues, the need to resource pre‐engagement and long‐term partnerships grows stressed in the reference panels as crucial to enable involvement (Appendix 6). We would urge that further contributions be made to the literature on how reciprocal projects with seldom heard groups have resulted in reforms and changes linked to the six BCW policy categories. Additional work should also expand and refine these programme theories by engaging with other seldom heard groups.

We recognize that there are limitations within this work in particular that an RRR is not a comprehensive search, review and synthesis of the literature. However, the methodological strength and process of engagement allowed for a broad engagement with seldom heard people and organizations representing them. The RRR process enabled us to capture local expertise via our six reference panels and the insights they shared captured valuable mechanisms that enhanced the richness of the review. RRRs can support PPI initiatives by producing programme theories of what works. This work contributes to a field where there has been little evidence of what works. It is evident from our developed programme theories that mechanisms and associated resources need to combine and interact to enable and sustain the reciprocal involvement of seldom heard groups in health and social care research.

## CONFLICT OF INTEREST

The authors declare that they have no competing interests.

## AUTHORS’ CONTRIBUTIONS

All the authors have made significant intellectual or practical contributions towards the development of the RRR. ÉNS drafted this paper, and all authors read, edited and approved the final manuscript.
