# Supplementary material for: Clarifying the mechanisms and resources that enable the reciprocal involvement of seldom heard groups in health and social care research: A collaborative rapid realist review process
Source: Health Expect. 2019 Feb 6;22(3):298–306. doi: 10.1111/hex.12865 (PMC6543157; doi:10.1111/hex.12865)
Supplement: Supplementary file 1 [file HEX-22-298-s001.doc]

**Appendix 1 Expert Panel Membership**

- Ms Joan O’Connor, Disability Federation Of Ireland;
- Ms Eleanor Dunn, Dublin Simon Community;
- Dr Cliona Loughnane, National Women’s Council of Ireland;
- Ms Vanessa Lacey, Transgender Equality Network of Ireland
- Ass Prof Maura Adshead, UL Engage, University of Limerick
- Ass Prof Amanda McCann, Conway Institute of Biomolecular and Biomedical Research, University College Dublin (UCD)
- Dr Sarah Morton, School of Social Policy, Social Work and Social Justice, UCD
- Ass Prof Veronica Lambert, School of Nursing and Human Sciences, Dublin City University
- Dr Cliona Ní Cheallaigh, Consultant in General Medicine and Infectious Diseases, St James’s Hospital & School of Medicine Trinity College Dublin
- Dr Éidín Ní Shé, School of Nursing, Midwifery and Health Systems, UCD
- Prof Thilo Kroll, School of Nursing, Midwifery and Health Systems, UCD

**Appendix 2 Key defined terms**

- **Seldom heard**: A term defined by [NHS involvement](http://www.nhsinvolvement.co.uk/connect-and-create/auto-generate-from-title/seldom-heard-groups) to ‘Describe groups who may experience barriers to accessing services or are under-represented in healthcare decision making. Traditionally, some of the groups identified in engagement activities include rural communities, black and minority ethnic (BME) groups, gypsies and travellers, lesbian, gay, bisexual and transgender, asylum seekers and refugees and young carers. However, in reality, teenagers, employees, people with mental health issues and many others may be considered as seldom heard, due to the fact engagement may not be straightforward’ Other population groups may also face marginalisation and exclusion from engagement, including people with disabilities, people with a rare disease, frail older people and people in institutional settings (e.g. care homes, prisons). Being ‘seldom heard’ indicates that existing structures, organisations and services that target their needs are not adequately enabling their voice to be heard via their current participation processes (Ryan et al., 2017)
- **Reciprocal Involvement/ Reciprocity** in the research relationship: Reciprocity implies give and take where there is a mutual negotiation of meaning and power. Reciprocity demands that the ‘research enables people to know and control their world by engaging participants from the start of the research planning and design’ (Baker et al., 2004: 182). Ref: Baker J. at al., Equality from Theory to Action, 2004 Basingstoke, Palgrave Macmillan.
- **RRR Context**: Something that can have an impact or block a mechanism. It may emerge from the intervention or by a broader ‘backdrop’ the intervention is operating.
- **RRR Resources:** Required to enable mechanism
- **RRR Mechanism**: a generative force that results in an outcome. Manifested as a reasoning or response to the resources or capabilities available by or embedded in an intervention.
- **RRR Outcome**: What happened intended or unexpected
- **RRR Programme Theory:** Outlines what is supposed to be done in policy or programme (theory of change).

**Appendix 3 Extraction Template**

**Appendix 4 Reference Panel Questions**

1. Background/ Context-Why is research in health and social care important?

2. How are/were members involved in health and social care research?

- - How are they invited? How to they share the process? Was there a payment made for expenses? Was the research when finished fed back to them?
  - What worked well/What did not work well?

3. How would they liked to be involved in research?

4. How would you like to be involved? What supports would we need to consider? (E.g. training/clear payment structures/psychological supports)

**Appendix 5: Extracted literature summaries (n**=20)

| **Author & Year** | **Authors country setting** | **Document Aim** | **Intervention Mechanism** | **Publication Type** | **Study design** | **Findings outcomes** | **Behaviour Change Wheel (BCW) Contextual Category** | **Extracted Mechanisms (M) & Resources (R)** |
| --- | --- | --- | --- | --- | --- | --- | --- | --- |
| *Adshead & Dubula 2016* | Uganda,  Tanzania, South Africa and Ireland | Reflective article on developing and sustaining an emancipatory research framework | Emancipatory Research (ER) | Academic | Reflective ER Using both research coalitions and learning partnerships | Common narrative presented by authors with number of concerns identified | Environmental Social Planning (ESP)  Guidelines  Fiscal Measures  Service Provision  Communication & Marketing (C&M) | **Incentivism** (M) Flexibility in funding of work packages to support community lead initiatives (R);  **Co-Design** (M) Created a ER checklist which was reviewed and modified (R);  **Environmental Restructuring (ER)(**M)- Enabling full participation of Sub-Saharan African partners by allocating additional library and professional networking opportunities (R);  **Service provision** (M) Reduce tension between stakeholders provide time at the beginning of the project for partners to outline their diverse perspectives as to what they want from the collaboration (R). |
| *Adshead et al., 2007* | Ireland | What was learned from the research partnership involving community and university partners in a emancipatory process to capture the experiences of Travellers and people with experience of the asylum process in respect to access of public services | ER | Grey Literature | ER Using both research coalitions and learning partnerships | Research used to develop an integration strategy in the county-Various community outputs created. | Guidelines  Fiscal measures  Service Provision  ESP | **Accessibility** (M) Surveys were translated and peer researchers were matched with students dependent on their needs around accessibility, literacy (R);  **Peer Research** (M) Recruited amongst the local community. Interviews were advertised locally via partners (R);  **Education** (M) Peer researchers and students were provided basic Training in social research (R);  **Incentivism** (M) Community researchers were paid for the work they undertook (cash or vouchers for asylum seekers) (R);  **Co-Design** (M) Time for Pre-Planning/Agenda setting with the research steering committee -Important to enable time for all partners to prioritise research focus (R). |
| *AVA 2016* | United Kingdom (UK) | Summary of a seminar to explore the themes of best practice around service user involvement for women facing multiple disadvantage. | Co-Design | Grey Literature | Seminar format | Best practice agreement | Service Provision  Regulation  ESP  Fiscal measures | **ER** (M) Separate Women only spaces are crucial (R);  **Co-Design** (M) Diversity of engagement and provide different options for involvement (R).  **Incentivism** (M) Specific Funding calls developed on co-design funders can play key role in creating opportunities (R);  **Education** (M) important to offer education for women to see how their involvement create change (R);  **Incentivism** (M) Offer food at sessions and provide childcare (R). |
| *Blackwell et al.,* 2017 | UK | Using Participatory action research between patients and ED staff to improve palliative care experiences | Participatory action research (PAR) | Academic | PAR process of co-design | Identified quality improvement priorities resulting in changed in ED palliative care. | Service Provision  ESP  Guidelines | **Co-Design** (M) Enable flexibility use innovative methods to enable and rolling recruitment flexibility (R);  **Ethical considerations** (M) due to the vulnerable nature of the participants follow on going and adopted guidelines (R). |
| *Bonevski et al*., *201*  *4* | Australia | Capture barriers to participation of socioeconomically disadvantaged groups in health research and strategies on how to increase engagement. | Systematic Review (SR) | Academic | SR | 31 studies identified-majority were based in the USA-a considerable number of barriers were identified. | Service Provision  ESP  Fiscal Measures  Guidelines  C&M | **ER** (M) Directly engaging and employing gatekeepers as project recruitment officers (R);  **Education** (M) Provide ongoing and flexible education that is accessible (R);  **Co-Design** (M) Using flexible methods (R);  **Incentivism** (M) Flexible payment options available (R);  **Co-Design** (M) Time for clarity and flexibility on data sharing (R);  **Co-Design** (M) Time and funding to enable long term partnerships (R). |
| *Brady et al.,*  *2016* | Ireland | To capture the process of people in recovery being involved in identifying and conducting their own research using a peer lead approach. | Community (PAR) approach | Grey literature | Community Participatory Action Research Approach | Gap for single parents especially women as there are other factors to consider such as childcare and less safe spaces. Stigma around recovery also needs to be challenged. | ESP  Fiscal Measures  Guidelines | **Incentivism** (M) Flexible funding to cover childcare (R)  **ER** (M) Safe spaces for women (R)  **Education** (R) Ongoing education on experiences of people in recovery to remove the stigma (R) |
| *Clayson et al., 2018* | UK | To report the findings from reflexive data collection on the evolving co-production research relationship between the two "worlds" of community and academia. | Co-Production | Academic | Reflexive data from perspectives of an intermediary community partner, academic partners, and community researchers on experiences of a series of coproduction projects. | Number of ‘clash points’ identified where the co-production methodology and procedure clashed with traditional research methods including; reasons why people would participate; achieving reach in regard to the research process; how knowledge was reciprocated; how the process contributed to people's recovery processes; how relationships were developed; risk in terms of people feeling uncomfortable or challenged by the process; and tensions between different co-producers. | ESP  Service Provision  Regulation | **Co-Design (**M) Risk is perceived differently by different stakeholders power relations need to be reformed in ethical policy (R);  **Peer-Researchers** (M) Creating an enabling space to develop capacity and confidence (R);  **Co-design (**M) Including an intermediary to enable and communicate activities between stakeholders (R). |
| *Couch et al., 2014* | Australia | Article presents key learnings in conducting research with highly marginalised young people | Co-production | Academic | 3 case studies presented | Identifies a number of issues in conducting research with highly marginalised young people | Service Provision  ESP | **Co-Design** (M) Method of engagement with highly marginalised young people must factor in time and resources to enable trust (R);  **Education** (M) to support researchers to understand the social context for which there are undertaking the research within (R);  **Co-design** (M) Use of community researchers enabled increased participation (R). |
| *Dawson et al., 2018* | UK | SR to characterise and critique the empirical literature on black and minority ethnic group-PPI involvement in health and social care research. | SR | Academic | SR | 45 studies identified, mostly USA based. Majority of studies illustrated involvement during the research design phase, and least in the data analysis and interpretation. | Guidelines  Service Provision  Legislation  ESP | **Accessibility** (M) provide time for all partners to explore innovative ways of embedding BME involvement (R);  **Accessibility** (M) Translation and culturally appropriate materials-designed and accessible for involvement (R);  **Co-Design** (M) to encourage the right type of involvement and that which reflects the population takes times (R). |
| Hernandez et al*., 2010* | UK | To improve understandings about how seldom heard groups and social care services can work together to establish inclusive involvement practice. | Co-design | Academic | Reflective data | A practice model is outlined to promote and institutionally embed participation. | Service Provision | **Peer-researchers** (M) process must be flexible and is commenced at inception (R);  **Education** (M) to enable ongoing participation and feedback (R). |
| *Holroyd-Leduc et al., 2016* | Canada | Engaging older adults living with frailty and their family caregivers in research and decision making and in health policy | Co-Design | Academic | Reflective data on a number of research co-production projects. | Number of factors needs to be considered to engage this vulnerable population. | ESP  Service Provision  Legislation  Fiscal Measures | **Education** (M) needed for researchers on diversity of engagement methods (R);  **Accessibility** (M) use web-based communication platforms such as SKYPE or online discussion boards (R);  **Co-Design** (M) Include intermediary partner in supporting co-production activities (R);  **Incentivism** (M) financial costs need to be considered or buy out time cover for carers (R);  **Co-Design** (M) Reform ethics as respectful engagement requires assuming competence as the default and addressing each individual accordingly (R). |
| *Kaiser et al., 2016* | USA | Diversifying participants in clinical research to eliminate health inequalities | Co-Production | Academic | Reflective case study data | Community Advisors on Research Design and Strategies (CARDS) to bring hard‐to‐reach populations into the research process and have sustained their participation. | ESP  Service Provision  Guidelines  Fiscal Measures  C&M | **ER** (M) meetings in community settings to provide feedback on the appeal, clarity, and accessibility of materials and processes used in clinical research (R);  **Accessibility (**M) Plain language guidelines (R);  **Co-Design** (M) Monthly fees to the community centres the also included costs for child care and transportation for community members to attend. (R);  **Co-Design** (M) resource intermediary partner in supporting co-production activities (R);  **Incentivism** (M) Flexibility in payment method (R);  **Incentivism (**M) Import to celebrate with members-meals, holiday treats and small tokens such as gift cards (R)  **Co-Design** (M) Supporting members of CARDS who applied for jobs by providing letters of reference providing CV templates (R).  **Education** (M) Create a forum to enable personal motivations for doing research is developed to overcome stereotypes of researchers as cold academics who use research participants to serve their own ends (R). |
| *Kauffman et al., 2013* | USA | Identify methods to engage hard-to-reach patients in patient-centred outcomes research. | Co-design | Academic | Reflective case study | To make patient-centred outcomes research more meaningful the focus should be on building and maintaining trust, which is achieved via pre-engagement with communities and continuous engagement of study participants and their communities. | Service Provision  ESP  Legislation  Guidelines  Fiscal Measures  C&M | **Co-design** (M) intermediary partner in supporting pre-engagement co-production activities before during and after the research process to enable feedback loop (R);  **ER** (M) Meeting in community setting as defined by community partners may be on the streets, hair shops community settings etc (R);  **Accessibility** (M) clear assessible language and include ongoing verbal consent (R);  **Education** (M) Ongoing education on what is meant by research (R);  **Co-design** (M) work in partnership to develop outputs relevant to community partners (R). |
| *Luchenski et al., 2017* | UK | To complete an evidence synthesis of health and social interventions for inclusion health of people with experiences of homelessness, drug use, imprisonment and sex work. | Peer-research | Academic | SR with peer engagement | The views of those who have experienced social exclusion can be used to guide practitioners and service intervention. | ESP  Legislation  Guidelines  Fiscal Measures  Service Provision  C&M | **Peer-researchers** (M) Ongoing and flexible training to enable participation (R);  **Incentivism** (M) provide food and flexible payment options such as cash or voucher (R);  **ER** (M) Host meetings in community settings (R);  **Accessibility** (M) diversity of community methods need to be included (R). |
| *Nguyen et al., 2010* | USA | Outline experience with a community based participatory research (CBPR) project in diverse communities of limited English proficient minorities. | CBPR | Academic | Reflective CBPR | A CBPR approach can make the process of collecting public health and health services data in hard-to-reach communities not only viable, but also highly successful. | Service provision  ESP  Guidelines  C&M | **Co-Design** (M) time to enable shared decision making in developing and implementing the study with all partners (R)  **Peer-Researchers** (M) employed from community partners to collect data (R);  **Education** (M) for researchers to be aware that  the level of detail required for peer-reviewed publication is not necessarily required for community reporting, and that for community partners, timely reporting is the more important than extensive statistical rigor (R);  **ER** (M) Time for researchers to be present with community partners to develop trust (R). |
| O'Reilly et al*., 2016* | Ireland | Addressing the gap of migrant’s perspectives not being included in the development of GP guidelines in how to communicate appropriately with hard to reach migrants. | Participatory Learning and Action (PLA) Approach | Academic | Reflective PLA | PLA Approach is powerful practical 'fit-for-purpose' methodology for enabling hard-to-reach groups to engage meaningfully and contribute with ease to academic research. | Service provision  ESP  Guidelines  Legislation | **Education** (M) ongoing education for peer researchers (R);  **Peer researchers** (M) recruited to collect data (R);  **Incentivism** (M) Specific Funding calls supporting a PLA approach (R). |
| *Rafie et al., 2015* | USA | To develop an educational program to increase breast cancer screening amongst African Americans. | Co-design | Academic | Reflective | Developed an effective Breast Health Research Champion training program for women interested in becoming advocates for breast cancer screening and research in their community. | ESP  Service Provision | **Peer Researchers** (M) recruited for data collection (R);  **Co-Design** (M) intermediary partner key to supporting co-design (R);  **Education (**M) Ongoing training provided for peer researchers (R). |
| *Richard et al., 2017* | Australia | Describes a methodological approach for the development and application of a relational model of engagement in a stepped wedge designed cluster randomised controlled trial (RCT). The purpose of the model is to embed engagement across the continuum of the trial which will test if an experience-based co-design intervention improves psychosocial recovery for people affected by severe mental illness. | Co-design | Academic | Reflective  RCT | Policy, organisational and service user data combined with evidence from the literature. Four perspectives support the theoretical framework of the relational model of engagement and this is organised around two facets: the relational and continuous. The relational facet is underpinned by relational ethical theories and participatory action research principles | ESP  Service Provision  Fiscal measures  C&M | **Peer Researchers** (M) recruited via community partners for data collection (R);  **Co-Design** (M) using innovation method by enlisting people with lived experience in the development of recruitment postcards by using their artwork to capture key messages for participation (R);  **Incentivism** (M) allocate funding to pay for peer researchers and for travel costs (R). |
| *Robinson et al., 2017* | Australia | Describes challenges for services seeking to engage parents who are subject to child protection measures and offers alternative. | Co-Design | Academic | Reflective | Case studies intervention program in a remote community illustrate that responsiveness and continuity of engagement of parents over time is a necessary. | ESP  Service Provision | **ER** (M) being present amongst the community to build trust and connections (R);  **Co-design** (M) build connections with local community organisations (R). |
| *Ryan et al., 2017* | UK | Outline if priorities among seldom heard groups is different from mainstream views and, if so, how can these differences best be understood? | Co-Design | Academic | Reflective | There were few differences in what participants thought was important in health care but considerable differences in their expectations that they might personally receive good care. Differences related to participants' previous experiences. The drug users group reported particularly poor experiences and low expectations of good care. | ESP  Service Provision  C&M | **Peer Researchers** (M) involved in data collection (R);  **Co-Design** (M) role of intermediary partner to support co-production (R);  **ER** (M) Engagement in the community locations identified by external partners (R). |

**Appendix 6:** Reference Panel Summaries (n=6)

| **Organisation and Mode of Engagement** | **Summary Points** | **Contextual Category (BCW)** | **Mechanisms (M) & Resources (R)** |
| --- | --- | --- | --- |
| ***Dub Simon****-Workshop 17/5/18* | 1. Important to have time to enable agenda setting -to allow clients to identify research rather than having research proposal/agenda set; Moving away from practice of researchers coming to organisation with set research question/focus developed.  2.. Don’t have it too top down -important that you ensure that there are peer partners involved at each stage;  3. Training and assistance-Stressed as being very important to link any involvement to recognised training or supported for employment or internships (DIT example -Volunteer Cert; Retail Cert and a new Social Enterprise Cert);  4. Payments-important to offer flexibility as often clients are in receipt of social welfare -often vouchers are more suitable.  5. Timeframes of engagement -Important that you don’t delay. Researchers need to follow up as soon as possible due to the transient nature of some clients.  6. Important to use creative and Inclusive methods: reflecting health literacy and important to engage in diverse manners | ESP  Fiscal Measures  Service Provision  C&M  Guidelines | **Education** (M) Provide ongoing accredited education to community partners to support capacity building (R);  **Peer-researchers** (M) involved in identifying research focus and data collection (R);  **Incentivism** (M) Flexibility in funding methods for peer-researchers (R);  **ER** (M) researchers must follow up quickly following engagement (R);  **Co-Design** (M) use a diversity of inclusive methods (R). |
| ***Pavee Point Traveller and Roma Centre****-Conference call 23/5/18* | 1. Important that all the research is peer lead by Travellers or members of the Roma Community from inception, delivery to feedback.  2. Consultation currently is often a tick box exercise. Most of the time Pavee Point are approached by researchers after they have received funding. The project is often not culturally appropriate and health literacy is not considered.  3. The organisation has to spend a lot of time refining the projects-this is both frustrating and time consuming done with no resources.  4. Really important mechanism is time (with resources) to scope our research  5. Funders have a key role to play to ensure calls are culturally appropriate  6. Stressed that any training needs to be culturally appropriated and it needs to be accredited so that it can be used for further education/employment pathways for members of the Traveller and Roma communities.  7. Need to include a diversity of outputs that are co-designed and is accessible-ensure resource for health literacy.  8. Psychological supports -very important as there are very high suicide rates amongst the community-needs to be resources in for both community members and Pavee point staff who may be key facilitators. | ESP  Fiscal Measures  Service Provision  C&M  Guidelines  Legislation  Regulation | **Co-Design** (M) early engagement from the start where services users identify research the focus from the start (R);  **Accessibility** (M) inclusive of a diversity of outputs that is co-designed (R);  **Incentivism**(M) Specific Funding calls supporting a culturally appropriate approach (R);  **Education** (M) ongoing and that is culturally appropriate and accredited (R);  **Psychological Supports** (M) available for community and NGO staff (R). |
| ***Transgender Equality Network****- Meeting 24/5/18* | 1. Early engagement is key to shape proposals. Need to be given notice very early of potential collaboration often its last minute and is very frustrating.  2. Capacity of NGO staff needs to be considered-They are key gatekeepers that filter research. TENI staff numbers are very small the time that it takes for staff to be involved is very time consuming and is done as an *add on* to current workloads-this includes recruitment of service users this can take extra time to ensure their participation and no use/include the same people for projects.  3. As NGO that is a key gatekeeper they would like to see more connection and partnership agreements with universities that is long term-move away from current ad hoc process.  4. Diversity in funding call should enable long term partnerships.  5. Including psychological supports on research projects is key mechanism. Often service users are isolated, unemployed, marginalised -needs to be resourced and also available for staff. | ESP  C&M  Service Provision  Fiscal Measures  Legislation  Regulation | **Education** (M) Provide ongoing education to community partners to support capacity building (R);  **Psychological Supports** (M) available for community and NGO staff (R);  **Incentivism** (M) Diversity in funding calls to enable long term partnerships (R);  **Co-Design** (M) early engagement from the start where services users identify research the focus from the start (R). |
| ***Longford Women’s Link-***Conference Call 25/5/18 | 1. Challenge in Longford is that we have small diverse communities living locally this means that the engagement takes time-no one size fits all.  2. Our women’s group is framed around peer support;  3. It takes time to develop trust -initial engagement via phone and then you could move on to a series of conversations to enable the research to be shaped;  4. Role of Longford Women’s Link-act as enablers to provide a trusting space;  5. Childcare is very important. Cost of childcare needs to be considered-and attached to the activity in the space of engagement.  6. Transport-For rural communities there is an assumption that people would find their way. A key way of control is to prevent women to partake in events-lots of women use taxies but very expensive. Very important that its included in costings;  7. Important that researchers develop long-term relationships with organisations;  8. Need to provide education on evaluation for the organisation so that we can understand the value of the research-often education & capacity building is overlooked. | ESP  Regulation  Service Provision  Fiscal Measures | **Co-Design** (M) based on long term partnership that utilises a diversity of engagement methods (R);  **Co-Design** (M) intermediary partner is key to develop trust (R);  **Education** (M) Provide ongoing education to community partners to support capacity building (R).  **Incentivism** (M) to include funding for logistics and costs for social care (R). |
| ***Centres for Independent Living*** *-Email Consultation 4/6/2018- 16/6/2018* | 1. Accessibility and support for example buildings and meetings rooms are not accessible, personal assistants, note takers, scribes may be needed and are not funded, and technology may be required and is not in place.  2. Mobility allowance to enable provision of accessible transport to and from meetings, funding to provide hours for personal assistants, note takers etc., funding to ensure technology is made available.  3. People running the research need to conduct an accessibility audit, not just of their building or meeting room, but of the type of research they are conducting and how that may need to change in order to include people with a disability. Then resources are required in order to support the individual through the research programme. | ESP  Fiscal Measures  Service Provision | **Accessibility** (M) audit of research project to ensure accessibility (R);  **Incentivism** (M) to include funding for logistics and costs for personal assistants etc (R). |
| **Disability Federation of Ireland***-*Conference call with staff members 18/7/2018 | 1.We are supposed to cover the costs to enable participation -No payment for transport, no offer for a scribe, translation, meeting is often held in an inaccessible place- -This has a power imbalance;  2. No opportunity to shape the agenda-often our inclusion is tokenistic where the impetus is often to fill a slot;  3. Practical stuff needs to be considered from the start. The reality of engagement needs to be considered e.g. recognise the role of personal assistants;  4. Important to enable feedback to inform participants –A communication plan from the start needs to be considered;  5. Diversity of involvement needs to be considered using innovative methods-so not to enable participation burnout;  6. Co-facilitators-costings need to be considered to enable involvement-that must be flexible;  7. Opportunity to use people with disabilities skills: Many are accredited from many courses and don’t have the opportunity to use what they have learned. Audit of participants as to what they are willing to contribute.  8. Rural engagement –What is needed to facilitate involvement? Costs and logistics need to be considered form the start.  9. Ongoing training is needed for funders on what is needed to enable engagement  10. Time and education for researchers to understand boarder context. | ESP  C&M  Service Provision  Fiscal Measures  Legislation  Regulation | **Co-Design (M)** early engagement from the start that is inclusive enabling services users to identify the research (R);  **Education (M)** to funders on the costs required of engagement to be included in funding calls (R);  **Co-Design (M)** undertake an audit of available skills within community partners and ensure they have opportunities to utilise them**;**  **Incentivism** (M) to include funding for logistics and costs for personal assistants (R). |
